# Reference database of teeth images from the Family Bovidae

**DOI:** 10.1038/s41597-022-01501-4

**Published:** 2022-07-11

**Authors:** Juliet K. Brophy, Gregory J. Matthews

**Affiliations:** 1 Department of Geography and Anthropology, Louisiana State University, Baton Rouge, LA USA; 2 The Centre for the Exploration of the Deep Human Journey, University of the Witwatersrand, Private Bag 3, Wits, 2050 Johannesburg, South Africa; 3 Department of Mathematics and Statistics, Loyola University Chicago, Chicago, IL USA

**Keywords:** Palaeoecology, Archaeology

## Abstract

Researchers typically rely on fossils from the Family Bovidae to generate African paleoenvironmental reconstructions due to their strict ecological tendencies. Bovids have dominated the southern African fauna for the past four million years and, therefore, dominate the fossil faunal assemblages, especially isolated teeth. Traditionally, researchers reference modern and fossil comparative collections to identify teeth. However, researchers are limited by the specific type and number of bovids at each institution. B.O.V.I.D. (Bovidae Occlusal Visual IDentification) is a repository of images of the occlusal surface of bovid teeth. The dataset currently includes extant bovids from 7 tribes and 20 species (~3900). B.O.V.I.D. contains two scaled images per specimen: a color and a black and white (binarized) image. The database is a useful reference for identifying bovid teeth. The large sample size also allows one to observe the natural variation that exists in each taxa. The binarized images can be used in statistical shape analyses, such as taxonomic classification. B.O.V.I.D. is a valuable supplement to other methods for taxonomically identifying bovid teeth.

## Abstract (summary)

**Table:** 

Measurement(s)	occlusal surface of bovid teeth were outlined
Technology Type(s)	GIMP
Factor Type(s)	Bovidae taxonomic identification
Sample Characteristic - Organism	Bovidae
Sample Characteristic - Location	southern Africa

## Background & Summary

Fossil remains from the Family Bovidae, such as antelopes and buffalo, are frequently used to reconstruct past environments^1–3^. Bovids reflect distinct ecological adaptations in terms of diet, habitat, water dependence, and seasonal migrations that vary according to their respective ecological niches. Widespread cooling in the late Miocene led to a major adaptive radiation of the bovids, and increasingly they began to exploit more open environments^4–6^. Thus, by approximately 4 Ma, bovids came to dominate the African fauna, replacing the previously abundant suids^7–9^. The current distribution of bovids extends across the African continent in myriad environments that differ significantly in proportions of wood and grass cover.

The importance of bovid remains to paleoanthropological research was established initially by Broom^10,11^ and Wells and Cooke^12^. This dependence has been expanded and now ranges from paleodietary studies and evolutionary trends to hominin behavioral patterns^13–15^. In addition, several studies have demonstrated that changes in the relative abundance of bovid taxa reflected in fossil assemblages are indicative of fluctuations in environmental conditions, as bovids appear to be particularly responsive to environmental changes^16–18^.

Bovid teeth, in particular isolated teeth, make up a majority of the southern African fossil record. Thus, bovid teeth, coupled with their ecological tendencies, are important sources of information for reconstructing the paleoenvironments associated with the fossil hominins. Taxonomic identification of fossil bovid teeth, however, is often problematic; biasing factors such as age and degree of wear complicate identifications and often result in considerable overlap in the shape and size of teeth. Traditionally, researchers rely upon modern and fossil comparative collections to identify isolated bovid teeth. However, researchers are somewhat limited by travel and the specific type and number of bovids housed at each institution. Here, we present B.O.V.I.D. (Bovidae Occlusal Visual IDentification) which is a repository of images of the occlusal surface of bovid teeth (~3900). The purpose of the database is to allow researchers to visualize a large sample of teeth from different tribes, genera, and species. The sample includes the three upper and three lower molars in multiple states of wear from the seven most common tribes in the southern African fossil record and the twenty most common species from those tribes. This design will help researchers see the natural variation that exists within a specific tooth type of a taxon and, with the current sample, help taxonomically identify extant and fossil teeth with modern counterparts.

## Methods

**Fig. 1:**
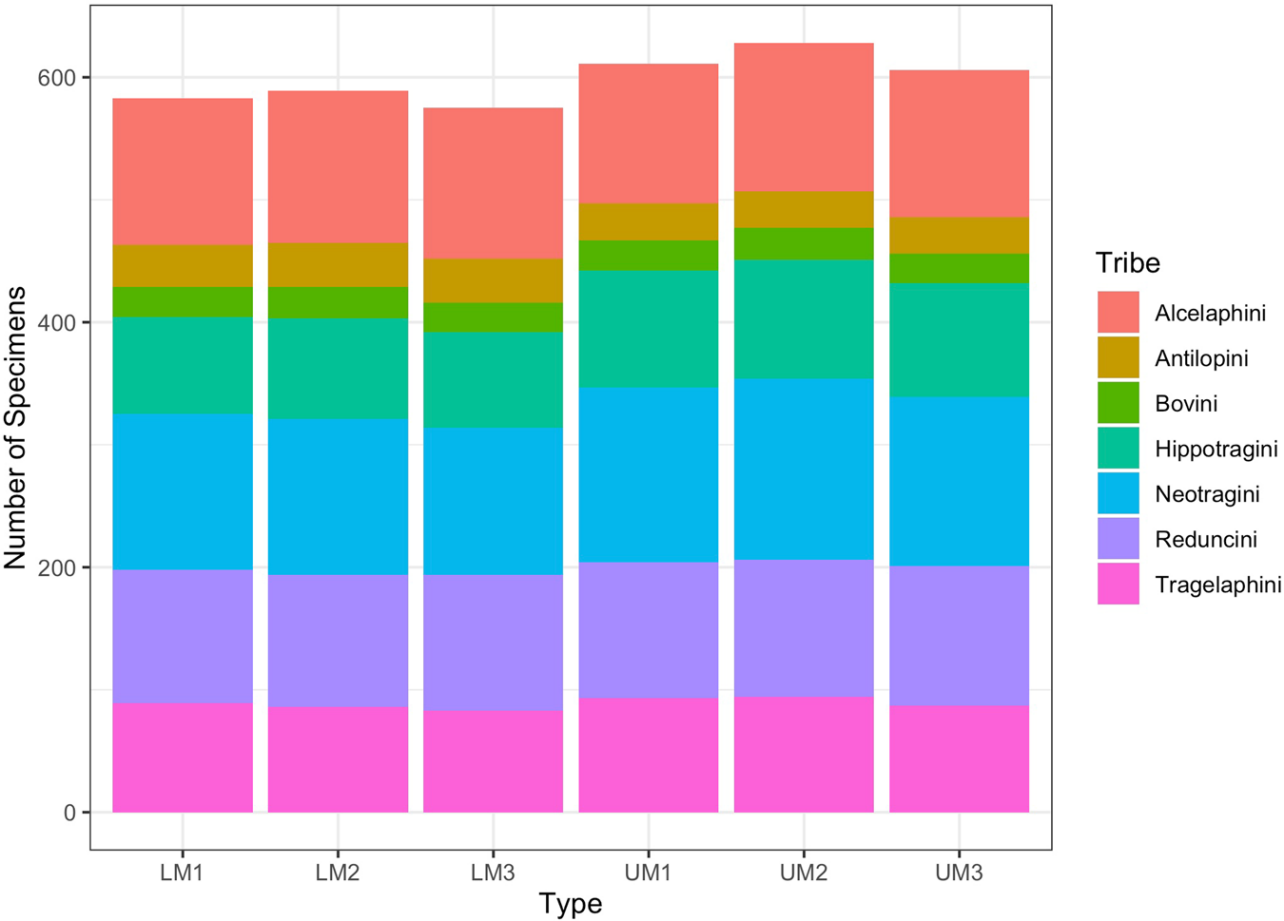
Visual summary of the data. Chart shows the distribution of tooth types by tribe. Note that some tribes have more species than other tribes (see Table 1).

Photographs of modern bovid teeth were obtained from four South African institutions: National Museum, Bloemfontein (NMB); Ditsong Museum (formerly Transvaal) (TM), Pretoria; and Amathole Museum (Amathole), King William’s Town. Images were also taken at the Field Museum (FM), Chicago, U.S.A. Table 1 and Fig. 1 present the bovids currently in the database. The bovids were wild shot, non-zoo specimens, according to the specimen box and/or the institution’s information spreadsheet.

**Table 1:** Extant bovid species in the database.

Tribe	Species List
Alcelaphini	
	*Damaliscus dorcas*
	*Alcelaphus buselaphus*
	*Connochaetes gnou*
	*Connochaetes taurinus*
Antilopini	
	*Antidorcas marsupialis*
Neotragini	
	*Oreotragus oreotragus*
	*Ourebia ourebi*
	*Pelea capreolus*
	*Raphicerus campestris*
Tragelaphini	
	*Tragelaphus scriptus*
	*Tragelaphus strepsiceros*
	*Taurotragus oryx*
Bovini	
	*Syncerus caffer*
Hippotragini	
	*Oryx gazella*
	*Hippotragus equinus*
	*Hippotragus niger*
Reduncini	
	*Redunca arundinum*
	*Redunca fulvorufula*
	*Kobus leche*
	*Kobus ellipsiprymnus*

**Fig. 2:**
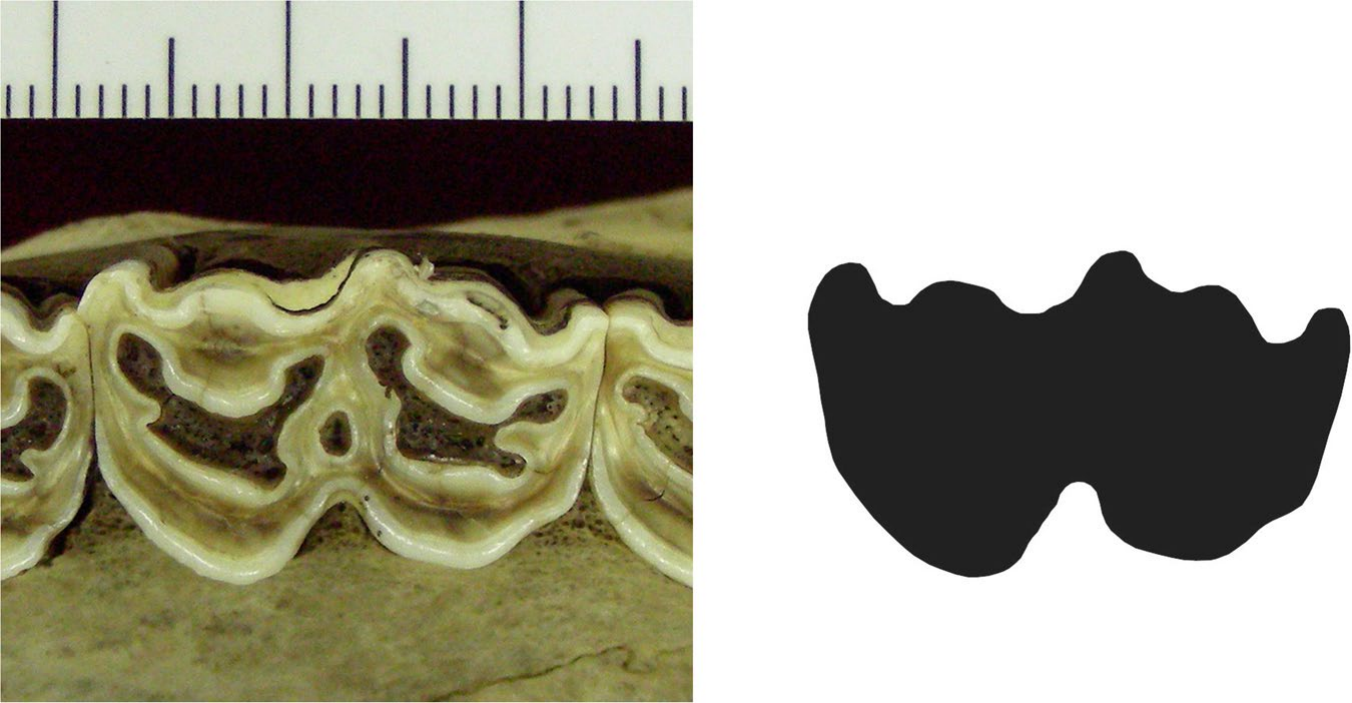
Example of a color (left) and bw photo (right). Specimen is DSCN2811, an upper second molar (UM2) *Alcelaphus buselaphus* NMB 12264.

Separate images were taken of the three molars from the upper and lower dentitions for each bovid specimen (see Fig. 2). All bovid teeth were photographed regardless of their level of attrition. A digital camera was positioned with a tripod directly above the occlusal surface of the tooth and leveled using a bubble level. Each cranium/mandible was situated so that the teeth were vertical and the occlusal surface could be seen clearly. The specimens were leveled and balanced using a bubble level, bean bags, and props. A stand with an adjustable clamp held a scale bar which was leveled, and placed directly next to the tooth at the height of the occlusal surface. Each picture was taken using the self-timer in order to assure that the camera was motionless. Pictures were taken at 300 megapixels resolution.

Whenever possible, the left side of the jaw was photographed. When teeth from the right side were used, the images were flipped horizontally in Adobe Photoshop® in order to make them left. The images were also cropped to highlight each specific tooth. Due to differences in the size of the bovid specimens, the distance of the camera from the tooth varied. Thus, each bovid tooth photo was processed in order to ensure that they are all at the same scale. Using the gridlines in Adobe Photoshop®, 1 centimeter on the scale bar in the image was matched to the program’s 1-inch ruler. This processing ensured that all teeth can be visualized and analyzed in the same orientation and at the same scale. Using the freeware GIMP^19^, the outlines of the teeth were obtained and converted into black and white images using the lasso function (Fig. 2).

## Data Records

This data set includes three main types of files: 1) Digital color images of the occlusal surface for each tooth in the data set, which have all been set to the same scale for easy comparison of size across image; 2) Black and white binary images extracted from the raw digital images containing the shape of the occlusal surface; 3) A meta-data file, which includes information such as taxonomic classification (e.g. tribe, genus, etc.), tooth type, side, etc. (details listed below).

This data is available in the Open Science Framework at 10.17605/OSF.IO/R5HSW, a data repository created by the Center for Open Science^20^. This repository contains all of the raw digital images, the black and white images, and the metadata file. In addition, the repository’s wiki includes a data dictionary. A dynamic version of the website is located at https://bovid.lsu.edu/.

Note that while the database is constantly updated, the repository on osf.io will contain “snapshots” of the database frozen at a particular time and new versions will be released when the database changes substantially. By freezing these versions, users will be able to better reproduce previous work and compare directly any potential analysis to a past analysis that used a particular version of the database.

## Technical Validation

One author collected all of the original color images. The pictures were processed and made into binary images at the Department of Geography and Anthropology, Louisiana State University. We aim to maintain B.O.V.I.D. with the highest quality data. All suggested data additions from outside researchers will be verified before combining with the dataset. We encourage users to report errors by emailing the corresponding author.

## Usage Notes

Researchers who use the database are asked to cite this publication. The database is open-access but if you would like to download the images and/or contribute your own images to the database, we request that you set up a free account with Open Science Framework and cite this publication.

## Data Availability

No code was used to generate this data.
